# Lead Poisoning

**Published:** 1902-08-09

**Authors:** 


### LEAD POISONING.

AN interesting discussion took place on the subject of lead poisoning, Dr. S. King Alcock, of Burslem, introducing the subject in a paper on "The advantages of periodical examination in dangerous trades." The cases to which he more particularly referred were those processes in the making of china which involved the use of lead. The points to be aimed at in periodical official examinations were the elimination of the unfit, the early detection of dangerous symptoms, and the accumulation of statistics. He thought that the economics of the question would be much altered and simplified if compensation wages were to be paid during enforced idleness after suspension. Certain workers were presented for examination who were inherently unfit to work in dangerous trades in consequence of marked debility or organic disease, and from an insurance point of view it was manifestly unfair to permit them to follow such an occupation when their state at entry was so obviously at fault.

Dr. Bond, of Poole, regarded anæmia as the most dangerous factor among female workers amongst lead. Starting with a diminished number of red blood cells and a corresponding enfeeblement of oxygenation, the influence of lead was entirely pernicious in such cases, and the end was apt to come with startling suddenness. Certain individuals were highly susceptible to the influence of lead, and its effects were not demonstrated by any obvious sign or signal until they suddenly became unconscious and died. In the case of girls with fresh complexions who became sallow and heavy and cachetic at their work their course was secure and imperative; but the fatal cases that had occurred in his experience seemed to have been of a nature that nothing short of periodic blood counts could have foretold.

Mr. W. H. Vickery, of Newcastle-on-Tyne, also bore testimony to the importance of a progressive anæmia as a reliable sign of dangerous impregnation with lead. He pointed out that every worker in white lead, no matter how careful, absorbed a certain amount of lead, and what the surgeon had to do was to determine when that absorption had passed the boundary line of safety. Blue line on the gums and muscular weakness were good corroborative signs, but if they were to suspend every worker with bad teeth it would be almost impossible for lead works to be carried on at all; and it had seemed to him that workmen who had neuritis were singularly free from other signs of lead-poisoning, and were very healthy-looking, and many of them, although their hands were distorted by paralysis, were very healthy in other respects, and had worked for years in lead without, as they put it, having had a day's sickness. Progressive anæmia was, however, a trustworthy sign, and one with which the worker could not interfere.

